# Three-dimensional visualization of brain tumor progression based accurate segmentation via comparative holographic projection

**DOI:** 10.1371/journal.pone.0236835

**Published:** 2020-07-30

**Authors:** Rania M. Abdelazeem, Doaa Youssef, Jala El-Azab, Salah Hassab-Elnaby, Mostafa Agour

**Affiliations:** 1 Engineering Applications of Laser Department, National Institute of Laser Enhanced Sciences “NILES”, Cairo University, Giza, Egypt; 2 Physics Department, Faculty of Science, Aswan University, Aswan, Egypt; Nicolaus Copernicus University, POLAND

## Abstract

We propose a new optical method based on comparative holographic projection for visual comparison between two abnormal follow-up magnetic resonance (MR) exams of glioblastoma patients to effectively visualize and assess tumor progression. First, the brain tissue and tumor areas are segmented from the MR exams using the fast marching method (FMM). The FMM approach is implemented on a computed pixel weight matrix based on an automated selection of a set of initialized target points. Thereafter, the associated phase holograms are calculated for the segmented structures based on an adaptive iterative Fourier transform algorithm (AIFTA). Within this approach, a spatial multiplexing is applied to reduce the speckle noise. Furthermore, hologram modulation is performed to represent two different reconstruction schemes. In both schemes, all calculated holograms are superimposed into a single two-dimensional (2D) hologram which is then displayed on a reflective phase-only spatial light modulator (SLM) for optical reconstruction. The optical reconstruction of the first scheme displays a 3D map of the tumor allowing to visualize the volume of the tumor after treatment and at the progression. Whereas, the second scheme displays the follow-up exams in a side-by-side mode highlighting tumor areas, so the assessment of each case can be fast achieved. The proposed system can be used as a valuable tool for interpretation and assessment of the tumor progression with respect to the treatment method providing an improvement in diagnosis and treatment planning.

## Introduction

Follow-up of malignant tumors progression after surgery is a critical and vital criterion to evaluate the current state of a patient with respect to a given treatment method such as radiotherapy and/or chemotherapy [1, 2]. Glioblastoma is one of the most common primary malignant tumors in humans. It is formed due to the accumulation of abnormal cells within the brain tissue that often grow and spread rapidly into other healthy tissue [3]. Glioblastoma is known to be aggressive, destructive and difficult to treat [2, 4]. Commonly in the literature [2, 4–6], the follow-up of tumor progression is realized by visual comparison between two or more magnetic resonance (MR) images or computed tomography (CT) scans captured at different time with or without highlighting the brain abnormalities. Accordingly, the segmentation of abnormal brain structures is significant not only for the diagnosis and medical analysis but also for the treatment planning and follow-up.

Over the past decades in industry and production, comparative methods which are based on holography have been developed for the assessment of fabricated products and comparing them with the standard products. Comparative holography was firstly proposed by Neumann in 1980 [7] to detect the differences between the microstructures of two different objects. Subsequently, this technique was improved by Fuzessy and Gyimesi [8] who applied a double reference beam method for the reconstruction and storage of the two objects. Moreover, Osten et. al. [9] proposed a method that permitted a direct holographic comparison of the differences in deformation or in contour between two objects (master and test objects), having different microstructures, located at different places. Whereas, the test object was illuminated by a coherent mask of the master object. Thus, remote shape control could be possible at any place without the physical presence of the master object. The coherent mask of the master object was only required which could be digitally sent using a telecommunication network. Consequently, the master object was projected by a spatial light modulator (SLM) allowing a direct comparison between the test and the master objects by highlighting the differences between them [10, 11].

Here, we propose an optical system based on comparative holographic projection, as an alternative method, to present the follow-up images in a side-by-side mode highlighting tumor areas for easily visualizing and assessing the tumor progression. It combines comparative digital holography, which is widely used in industry but is not used in the medical field, and holographic projection. Holographic projection is a method that provides three-dimensional (3D) information about an object without the need for special eyewear. Unlike the existing traditional photography that evaluates only the intensity distribution of a test object, computer-generated hologram (CGH) has been generated to permit direct access of both intensity and phase of the object field providing the complete 3D information [12–16]. Despite the generated CGH is a 2D distribution, it encodes the 3D information of the object [15–20]. Based on the diffraction theory, the calculated 2D CGH can be encoded in different ways for effective optical reconstruction to provide the complete 3D information of the recorded object. Commonly, SLMs had been used to reconstruct CGHs in real-time to get the original object floating in the air or displayed on a screen [21–27]. SLMs are dynamic devices that provide a direct method for manipulating the incident light by means of the displayed CGH. Thus, a predefined light distribution was generated at the observation plane [28, 29]. Due to the technical characteristics of the SLM such as the pixelated nature, the limited number of pixels, and the fill factor (less than 100%), there is undesirable effects at the observation plane, for instance, an envelop modulation, a DC term and replicated diffraction orders, these artifacts can be compensated obviously during the design process of the CGH and/or the optical reconstruction [25, 26, 28–35]. In addition to the previously mentioned artifacts, there is arising speckle noise that deteriorates the projected image quality in the observation plane which is a result of the coherent light illumination. This effect can also be compensated using different multiplexing techniques [13, 36]. Thus, the combination of CGH with the SLM allows a dynamic holographic projection in real-time with high efficiency and low power consumption [18, 22, 37–39].

Our proposed comparative holographic projection system presents two follow-up MR exams for different glioblastoma patients alongside enabling fast assessment of each case. To achieve that, first, the brain tissue and the tumor areas were accurately segmented from the two MR exams using the fast marching method (FMM) which was implemented on a computed pixel weight matrix based on an automated detection of the initialized target points. Then, two CGHs (one for the brain tissue and the other for the tumor area) were calculated for each MR exam based on an adaptive iterative Fourier transform algorithm (AIFTA). Additionally, a speckle reduction method based on temporal multiplexing of spatial frequencies was applied to minimize the speckle noise in the optical reconstruction. Furthermore, the calculated phase holograms were manipulated to:

1. allow the 3D visualization of the tumor: after completing chemi-radiation therapy (CRT) and at the progression.
2. avoid the overlapping of the follow-up images in the reconstruction plane.
3. highlight the detected tumor areas for efficient visualization in the reconstruction plane.

Finally, the manipulated phase holograms were reconstructed optically using a reflective phase-only SLM. Thus, the visual comparison could be fast achieved providing an improvement in diagnosis and in treatment planning.

In the following section, the segmentation method that was used to extract the brain tissue and the tumor area is illustrated. Consequently, the method used to generate CGHs are presented. Finally, the experimental setup and the results are discussed.

## Segmentation of brain tissue and tumor areas

The MR datasets used in this study were accessed from the brain tumor progression collection of the cancer imaging archive (TCIA) database [40, 41]. TCIA provides an open access resource to reinforce research development utilizing the advanced medical imaging of cancer. The datasets of the brain tumor progression were acquired from twenty glioblastoma patients treated by surgery and basic concomitant CRT followed by adjuvant chemotherapy. Each patient had two MR exams acquired: within ninety days after completing CRT and at the progression state which was based on the integration of the clinical performance and/or imaging outcomes.

**Fig 1:**
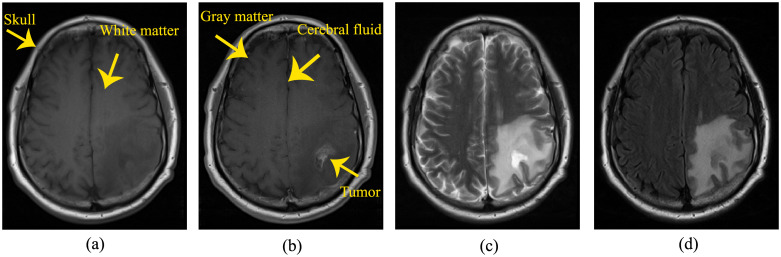
Four MR images of a glioblastoma patient with an irregular lesion in the parietal lobe of the left hemisphere. (a) T1 weighted, (b) T1 weighted post-contrast agent, (c) T2 weighted, and (d) T2 weighted FLAIR.

The datasets are in DICOM format and contain four series of T1-weighted, T1-weighted post-contrast agent (in which gadolinium contrast agent was utilized to increase the contrast between healthy tissue and lesion areas), T2-weighted and T2-weighted FLAIR modalities. Fig 1 illustrates the appearance of the brain tissue (that is surrounded by skull and consists of gray matter, white matter and cerebral fluid), and the tumor area in four MR images of a patient suffering from glioblastoma. It shows an irregular tumor in the parietal lobe of the left hemisphere. As shown in Fig 1, the tumor area is hyperintense and the edema around the tumor is identified in the T2-weighted and T2-weighted FLAIR in comparison with the T1-weighted pre- and post-contrast agent images. On the other hand, the tumor borders are well enhanced and conspicuous in the T1-weighted post-contrast agent image. Therefore, the segmentation and visualization in this study were performed on the T1-weighted post-contrast agent series as it is the best series to display the tumor area and its boundaries. It should be clarified that the T1-weighted post-contrast agent series of the datasets, after completing CRT and at the progression state, were co-registered and overlaid as a pre-processing step for assessing the brain tumor progression. Generally, an MR exam was acquired in more than one slice leading to image sequence with size *m* × *n* × *k*, where *m* and *n* are the numbers of rows and columns respectively in an image and *k* is the slice number. For the results of this study, the datasets of three glioblastoma patients were used. Detailed properties of the three patients’ datasets are presented in Table 1.

**Table 1:** Properties of patients’ axial T1 weighted post-contrast agent series.

	Case 1	Case 2	Case 3
*Image* *size* (*m* × *n*)	320 × 260 pixels	512 × 512 pixels	512 × 512 pixels
*Number* *of* *MR* *slices*	25	22	24
*Slice* *thickness*	5 *mm*	5 *mm*	5 *mm*
*Slice* *separation*	6.5 *mm*	6.5 *mm*	6.5 *mm*
*Pixel* *spacing*	0.6875 *mm*	0.4297 *mm*	0.4297 *mm*
*Magnetic* *field* *strength*	3 *T*	1.5 *T*	1.5 *T*
*Slices* *containing* *tumor*	16–19	15–18	12–15
*Tumor* *location*	Parietal lobe	Frontal lobe	Frontal lobe

Although manual segmentation of brain abnormalities from MR exams is an exhausting task for radiologists, it usually helps the physicians to put the right diagnosis. Thus, several methods have been developed for accurate segmentation or evaluation of brain tumors using different image processing techniques. For example, Diaz et al. [42] introduced an automated brain tumor segmentation method based on multi-thresholding and morphological operations by dilation. The method was applied to four MR modalities for sixteen real tumors to find the edema and gross tumor volume. It was demonstrated that the method was efficient, with the average Dice similarity coefficient of 0.81 in segmenting edema and 0.85 in segmenting gross tumor volume, and fast, with an average time of 49.18 sec for segmentation. Moreover, the authors in [43] segmented the brain tumor area form MR exams using thresholding and morphological operations. Then, the centroid, area and perimeter features of the tumor area were extracted. Four different methods, Otsu, k-means, fuzzy c-means and thresholding, to segment the brain tumor from MR images were provided in [44]. It had been mentioned that the thresholding and k-means methods took the least and highest computation time respectively while the fuzzy c-means method had the highest accuracy. In [45] fuzzy c-means clustering was applied where the MR image was labeled into three regions according to image intensity. Then, the tumor area was extracted from the rest of the segmented regions by calculating their areas and applying circularity as a criterion. Although, the circularity as a criterion is a poor feature to satisfy tumors. Their results were verified by comparing the segmented tumor with manually segmented ground truth by calculating the average Dice similarity coefficient (the obtained value was 0.729). An improved Sobel edge detection algorithm for brain tumor segmentation of MR image was proposed by Asra Aslam et. al. [46]. Since the extracted edges were not always close to the tumor area, they applied a closed contour algorithm to the detected edges for extracting the tumor from the MR image. However, the method should be modified to increase the segmented region area and decrease the region boundary thickness. As provide in [47], the authors used Otsu’s segmentation to extract the tumor region form MR exams after applying a 2D-adaptive filter to enhance the image quality. Then, morphological operations by erosion and dilation were applied to remove the skull boundaries and extra noise caused by segmentation. Based on the tumor size, it was classified into benign and malignant.

Unfortunately, most of the developed segmentation methods focused on the segmentation of brain tumors regardless of the brain tissue. While, a key step in this study was the choice of an appropriate image processing technique that could segment the brain tissue along with tumor areas from the MR exams with high accuracy. Therefore, an accurate and fast segmentation algorithm based on the fast marching method that was developed by James Sethian [48] was applied to segment the brain tissue and tumor area. The FMM is the fastest and the most efficient algorithm to estimate the first arrival [49]. Moreover, it is an accurate, stable, and computationally efficient when applied to image segmentation for 2D and 3D shape recovery [48, 49].

To increase the segmentation accuracy, the FMM was implemented on a computed pixel weight matrix derived from gray-level intensity differences which is discussed in the following subsection.

### Pixel weight matrix computation

For each pixel in the MR image, the intensity difference between this pixel and an initialized target point or the mean of the intensity values of initialized target points) within the segmented region of interest (brain tissue or the tumor area) was computed. Because of the purpose of automation, the initialized target points within the brain tissue were set using an algorithm based on Canny edge detector [50] which is the most powerful method that can detect true edges without being fooled by noise. Significantly, it can only preserve weak edge points in the image if they are eight-connected to predefined strong edge points. The canny edge detector method is summarized as follows [51]:

1. The image is smoothed with a Gaussian filter to reduce noise.
2. The local gradient, which is calculated from the derivative of the smoothed MR image, and the gradient direction for each pixel in the image are computed.
3. Only the image pixels whose strengths are locally maximum in the gradient direction are defined to be edge points. These edge points cause ridges in the gradient magnitude image. By tracking along the top of these ridges and setting to zero all pixels that are not on the ridge top, a thin line is formed.
4. Two thresholds are applied to the ridge pixels. Whereas the ridge pixels whose values are greater than the higher threshold are defined as strong edge points. Besides, weak edge points are defined for the ridge pixels with values between the lower and higher thresholds. While, all ridge pixels below the lower threshold are disregarded.

The proposed algorithm was implemented to set the brain tissue initialized target points by applying the canny edge detector two times to the MR image with two different high thresholds. Subsequently, the initialized target point within the tumor area was automatically selected as the point of the highest intensity within the MR image after skull stripping (i.e. from the segmented brain tissue).

After automatically defining the initialized target point/points, a weight value is given to this pixel according to its gray-level difference. It must be declared that the pixel weight matrix values are inversely related to the gray-level difference values.

### The fast marching method

The fast marching method is an approach implemented to solve boundary value problems when applied to image segmentation [52]. It considers the propagation of a front over a surface under a speed function *F*(*x*, *y*) defined on a surface over arrival time *T*(*x*, *y*) at point (*x*, *y*). When *F*(*x*, *y*)>0, *T*(*x*, *y*) function satisfies the Eikonal equation (Eq (1)) [53] which is a first-order non-linear partial differential equation encountered in wave propagation problems. While for *F*(*x*, *y*)<0 at any points (*x*, *y*), the FMM cannot find a solution for *T*(*x*, *y*). That is why a point can only be visited once. Moreover, the propagation stops when the speed approaches to zero [48]. Generally, the FMM aims to find a solution value for the function *T*(*x*, *y*) that describes the front propagation over time whereas the speed function is inversely proportional to the gradient of the arrival time as [48]:

$$\begin{array}{c} \left|\nabla T(x,y)\right|=\frac{1}{F(x,y)}. \end{array}$$ (1)

In a simple illustration, the FMM is a region growing algorithm in which an initial front is defined by choosing one or grid of seed points (*x*, *y*), tagged as narrow band points, whereas the function *T*(*x*, *y*) is initiated by zero. For each narrow band point, the arrival time *T*(*x*, *y*) is calculated depending on a threshold condition (*H*) for four neighborhood (trial) points (one point is away from this narrow band point). Consequently, the trial point with the smallest value of *T*(*x*, *y*) is updated into an accepted point and removed from trial points. Then, for each newly accepted point, its four neighborhood (trial) points are selected and the marching process is repeated. Finally, when the speed approaches to zero, the marching process stops and the segmented regions are obtained from the accepted points [48]. Therefore, the FMM approach allows only the propagation through one direction from smaller values of *T*(*x*, *y*) to larger values (i.e. from higher to lower speed).

## Holograms generation

The proposed hologram generation method could be regarded as a two step process. In the first step the hologram is generated using the well-known iterative Fourier transform algorithm (IFTA) [54], while in the second step we added a special hologram modulation. Both steps will be discussed in the following in more details.

### Phase-only holograms computation

Let us consider two parallel planes, one is the object/reconstruction plane where the object is placed and the other is the SLM plane where the hologram is generated. The projection which is used to propagate the wave field from one plane to the other consists of Fourier transformation (F) for forward propagation and inverse Fourier transformation ($F^{-1}$) for backward propagation. At each plane one intensity constraint is applied. At the SLM plane, it is assumed a plane wave illumination which means that the amplitude here is homogeneous and equal 1, everywhere. At the object plane, the original object amplitude that is calculated by taking the square root of the intensity replaces the obtained one after the implementation of $F^{-1}$. The whole discussed process is called IFTA [54].

Simply, the iterative process is started with a complex-valued object guess $U_{0}^{\left(0\right)}$ given by:

$$\begin{array}{c} U_{0}^{\left(0\right)}=\sqrt{I}.\exp\left(i\phi_{0}^{\left(0\right)}\right), \end{array}$$ (2)

where *I* denotes the intensity of the input object and $\sqrt{I}$ is its amplitude, and $\phi_{0}^{\left(0\right)}=0$ is the initial phase guess. For determining the diffracted field $U_{h}^{\left(n\right)}$ at the hologram plane, the complex-wave field $U_{0}^{\left(0\right)}$ is forward propagated to the SLM plane using the fast (F). Thus, the wave field at the SLM plane at *n*^*th*^ iteration can be expressed as:

$$\begin{array}{c} U_{h}^{\left(n\right)}=F\left\{U_{0}^{\left(n-1\right)}\right\}, \end{array}$$ (3)

where *n* represents the current iteration number. At the SLM plane, an amplitude constraint is applied (a plane wave illumination, i.e. *A* = 1), while the phase of the propagated wave field $\phi_{h}^{\left(n\right)}$ is kept unchanged. Thus, Eq (3) can be rewritten in the form:

$$\begin{array}{c} U_{h}^{\prime (n)}=\frac{A}{|U_{h}^{\left(n\right)}|}U_{h}^{\left(n\right)}=A.\exp\left(i\phi_{h}^{\left(n\right)}\right), \end{array}$$ (4)

where $\phi_{h}^{\left(n\right)}=arg\left\{U_{h}^{\left(n\right)}\right\}$ and arg is the complex argument of the complex number. Eq (4) explains mathematically how the intensity constraint at the SLM plane is applied. Here, dividing the complex function $U_{h}^{\left(n\right)}$ by its amplitude ($|U_{h}^{\left(n\right)}|$) leaves only the phase information which is multiplied with the plane wave illumination (*A* = 1).

Then, the back propagation to the object plane is achieved by applying ($F^{-1}$) as:

$$\begin{array}{c} U_{0}^{\left(n+1\right)}=F^{-1}\left\{U_{h}^{\prime (n)}\right\}. \end{array}$$ (5)

At the object plane, the original object amplitude $\left(\sqrt{I}\right)$ constraint is applied, in analogy to Eq 4, thus:

$$\begin{array}{c} U_{0}^{\prime (n+1)}=\frac{\sqrt{I}}{|U_{0}^{\left(n+1\right)}|}U_{0}^{\left(n+1\right)}=\sqrt{I}.\exp\left(i\phi_{0}^{\left(n+1\right)}\right). \end{array}$$ (6)

**Fig 2:**
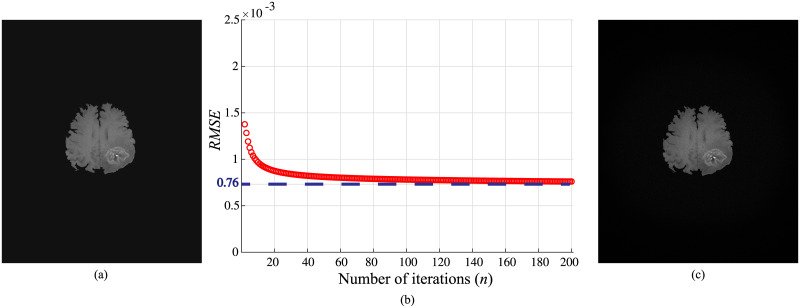
(a) Original image after zero-padding (the image size is 7680 × 4320 pixels), (b) *RMSE* as a function of number of iterations *n*, and (c) the numerical reconstruction of the MR image in (a) after *n* = 200 iterations. Note that, the dynamic range of the images (a) and (c) is varying from 0 to 1.

The phase $\left(\phi_{0}^{\left(n+1\right)}\right)$ is kept unchanged at this plane as well. The process is continued until there is no change in the phase is observed $(\phi_{h}^{\left(n+1\right)}-\phi_{h}^{\left(n\right)})<\epsilon$, where *ϵ* is the phase difference at the convergence of the algorithm. To monitor the quality of the numerical reconstructed image, the root mean square error (*RMSE*) was used. Fig 2(b) shows the *RMSE* of the zero-padded MR image in (a) as a function of *n*, where the total number of iterations is *n* = 200. The *RMSE* decreases gradually as the number of iterations increases, it can be noticed that the error stabilizes to be 7.6 × 10^−4^ after *n* = 162 iterations which corresponds to a phase difference of convergence *ϵ* = 0.05 radians. Thus, *ϵ* corresponds to a standard deviation smaller than 1% and was used as a stopping criterion, and this gives satisfying reconstruction results. Therefore, the exact computation time required for the design of the phase hologram was determined. The fast Fourier transform (*FFT*) implementation using Matlab requires *N*log_2_ *N* floating point operations to compute for an image size (*N* × *N*), where *N* represents the number of rows and columns. In each iteration of an IFTA, four *FFT*2 were implemented. Based on the power of the central processing unit (CPU)/ the graphics processing unit (GPU) used, the time required to arrive the convergence was calculated. In our experiment, the calculation time to arrive the convergence of the algorithm was 492 sec using Core i5-3210 CPU 2.5 GHz with 4 GB RAM, Matlab2018a. However, this time could be significantly decreased in milliseconds range if the approach was paralyzed and implemented in a GPU. The numerical reconstructed MR image after the convergence of the algorithm is shown in Fig 2(c).

### Holograms modulation

Here the second step of the proposed method is discussed. Note that, we referred to the IFTA (the first step) and the hologram modulation done in the following as the adaptive iterative Fourier transform algorithm (AIFTA). The proposed step allows the modulation of the calculated phase holograms to represent two different reconstruction schemes. In the first one, different layers containing the tumor areas that was segmented from the same brain tissue are selected for the visualization purpose (3D tumor map). In this case, the calculated holograms are modified by; i) chirp functions to imitate the 3D effect and ii) linear phase ramps to separate the tumor areas from the different layers across the reconstruction plane. In the second scheme, for a single slice, the brain tissue and/or tumor area is compared after completing CRT and at the progression. In such a case, the calculated holograms are only modified by linear phase ramps to avoid the overlapping at the reconstruction plane. In both schemes, all calculated holograms are superimposed in a single 2D hologram to be displayed on the reflective phase-only SLM.

#### Holographic 3D representation of the tumor

In this subsection, the modulation process of the first scheme to display different axial slices of a brain tumor is discussed. For this purpose, four partitioned MR slices of tumor representing four different axial layers are considered. The separation between each layer and the subsequent layer is 6.5 mm. Four digital holograms are calculated using IFTA, where each hologram is corresponding to one of the four partitioned MR layers. The holograms are modulated with four chirp and ramp functions. The modulation of the generated holograms with a quadratic chirp functions, i.e. transfer function of propagation ($\chi_{z_{s}}$) [55] can be achieved by multiplying the hologram with the transfer function ($\chi_{z_{s}}$). Then, the result is multiplied with a linear phase ramp (*R*_*z*,*s*_). Thus, the outcome complex amplitude hologram can be expressed as:

$$\begin{array}{c} U{”}_{h,s}=U_{h,s}^{\left(n+1\right)}\cdot \chi_{z_{s}}\cdot R_{z,s}, \end{array}$$ (7)

where *s* refers to the slice number and it takes the values of *s* = [1, 2, 3, 4], *z*_*s*_ is the propagation distance between two subsequent layers. The transfer function ($\chi_{z_{s}}$) and the phase ramp (*R*_*z*,*s*_) in the paraxial approximation are given by:

$$\begin{array}{c} \chi_{z_{s}}=\exp[ik\frac{\lambda^{2}}{2}z_{s}{\left|\vec{v}\right|}^{2}],\;\wedge \;R_{z,s}=\exp(ik[\sin\left(\alpha_{s}\right)\cdot v_{i}+\sin\left(\beta_{s}\right)\cdot v_{j}])\;, \end{array}$$ (8)

here *k* is the wave number, $\vec{v}=\left(v_{i},v_{j}\right)$ is a 2D vector across the SLM domain. The phase ramp (*R*_*z*,*s*_) is analogue to tilt the SLM with the angles *α*/2 and *β*/2 in the *v*_*i*_ and *v*_*j*_ directions. These two angles can be defined by the distance *z*_*s*_ between the SLM plane and the reconstruction plane and the required shift $(\Delta {\vec{u}}_{s}$) to laterally separate the tumor slices, thus sin(*α*_*s*_) = Δ*u*_*i*_/*z*_*s*_ and sin(*β*_*s*_) = Δ*u*_*j*_/*z*_*s*_. Eq (8) indicates that $\chi_{z_{s}}$ as well as *R*_*z*,*s*_ are pure phase functions which can be realized by means of the SLM.

After generating the four modified holograms, the final complex amplitude (*U*_*F*_) is calculated by complex addition of all holograms as $\left\{\sum_{s=1}^{4}U{”}_{h,s}\right\}$. To define the phase distribution (*ϕ*_*F*_) that is displayed on the SLM, the arg{*U*_*F*_} is calculated according to:

$$\begin{array}{c} \phi_{F}=\arg\{\sum_{s=1}^{4}U{”}_{h,s}\} \end{array}$$ (9)

**Fig 3:**
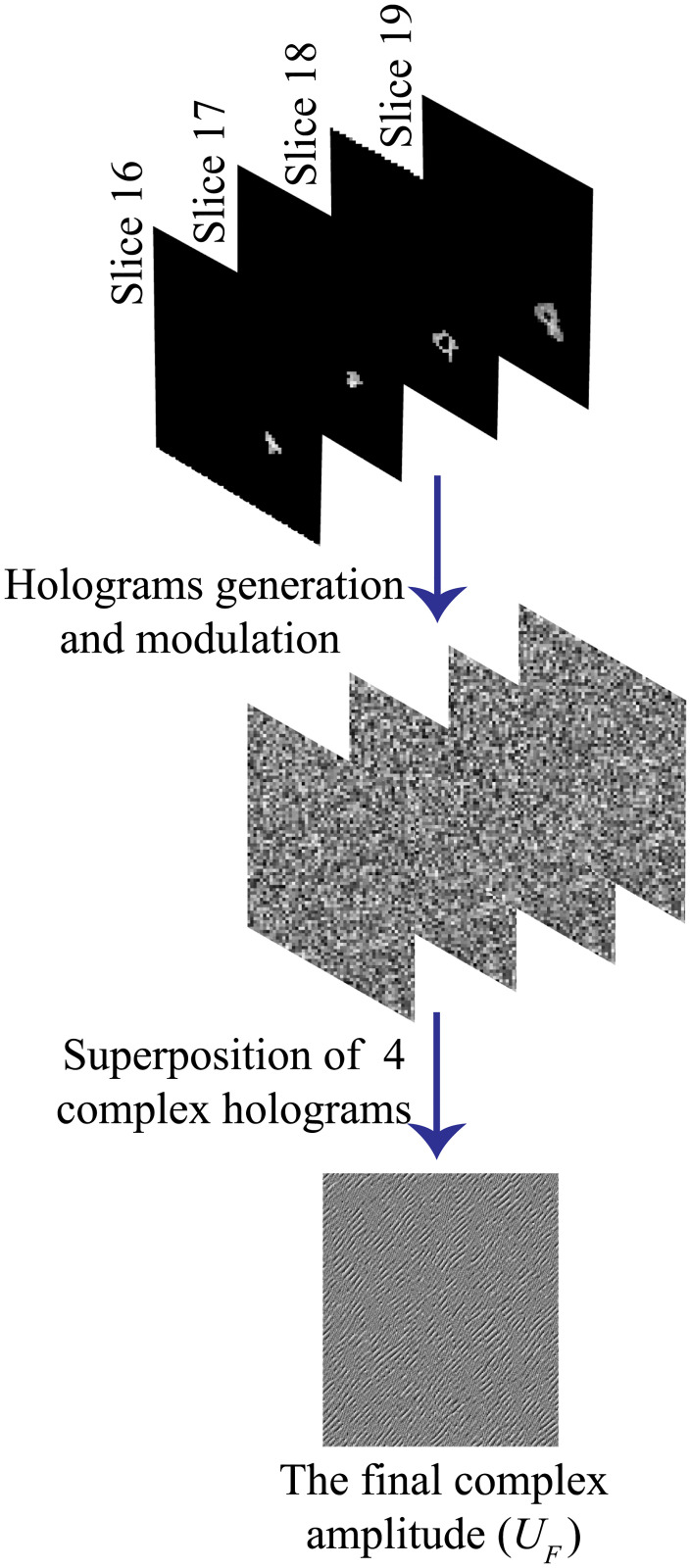
Diagram of AIFTA for tumor’s 3D computer-generated hologram calculations.

Since we are generating Fourier phase holograms, the modulation given in Eq (7) will turn into a convolution with a shifted copy of the impulse response (*ψ*_*z*,*s*_) after making a Fourier transformation to obtain the reconstruction plane. Accordingly, Eq (7) can be rewritten as:

$$\begin{array}{c} \sum_{s=1}^{4}F\{U{”}_{h,s}\}(\frac{\vec{u}}{\lambda f})=\sum_{s=1}^{4}F\left\{U_{h,s}^{\left(n+1\right)}\right\}(\frac{\vec{u}}{\lambda f})⊗\psi_{z_{s}}(\frac{\vec{u}}{\lambda f}-\frac{\Delta {\vec{u}}_{s}}{\lambda f}), \end{array}$$ (10)

where F denotes the Fourier transform applied by means of a lens with a focal length of *f*. This convolution is responsible for; i) the out of focus and ii) the shift $\Delta {\vec{u}}_{s}$ across the reconstruction plane. The diagram shown in Fig 3 is a simple illustration to the modulation process of the first scheme which is proposed to display sequential axial slices of a brain tumor.

#### Comparative holographic projection algorithm

For studying the second scheme, two MR slices were considered for each patient, after completing CRT and at the progression state. For each MR slice, two extended phase holograms were calculated, one for the brain tissue (*U*_*I*_) and the other for the tumor area (*U*_*T*_) using the IFTA. The modulated phase hologram (*ϕ*_*c*_) of the first MR slice (after completing CRT) is given by:

$$\begin{array}{c} \phi_{c}=\arg\left\{U_{h,s=1}\right\}=\arg\left\{U_{I}\right\}+\gamma \arg\left\{U_{T}\right\}. \end{array}$$ (11)

and the complex hologram ($U_{h,s=1}^{{}^{\prime }}$) of the second MR slice (at the progression state) is given by:

$$\begin{array}{c} U_{h,s=1}^{{}^{\prime }}=U_{I}^{{}^{\prime }}+U_{T}^{{}^{\prime }}. \end{array}$$ (12)

Thereafter, the complex amplitude of the second MR hologram ($U_{h,s=1}^{{}^{\prime }}$) is modulated only with a phase ramp *R*_*z*,1_ to separate the follow-up images in the observation plane as:

$$\begin{array}{c} U_{h,s=1}^{{}^{\prime \prime }}=U_{h,s=1}^{{}^{\prime }}\cdot R_{z,1}, \end{array}$$ (13)

where *R*_*z*,1_ is the pure phase function that is given by Eq (8). Then, the phase hologram (*ϕ*_*p*_) at the progression state can be written as:

$$\begin{array}{c} \phi_{p}=\arg\left\{U_{h,s=1}^{{}^{\prime \prime }}\right\}=\arg\left\{U_{I}^{{}^{\prime }}\right\}+\gamma \arg\left\{U_{T}^{{}^{\prime }}\right\}, \end{array}$$ (14)

where *γ* in Eqs (11) and (14) is a constant which is 2, used to modulate the intensity of the tumor area for highlighting it in the reconstruction plane. The final phase distribution (*ϕ*_*F*_) to be displayed on the SLM is calculated by adding 2*π* modulo of the phase distributions of the two MR exams as:

$$\begin{array}{c} \phi_{F}=\phi_{c}+\phi_{p}\;. \end{array}$$ (15)

Displaying such a phase hologram (*ϕ*_*F*_) on the SLM and illuminating it with a plane wave generates (across the back focal plane of a lens) the reconstructed follow-up images arranged in a side-by-side mode and highlighting certain features, i.e. in our case, the tumor area compared with the brain tissue.

## Experimental setup

**Fig 4:**
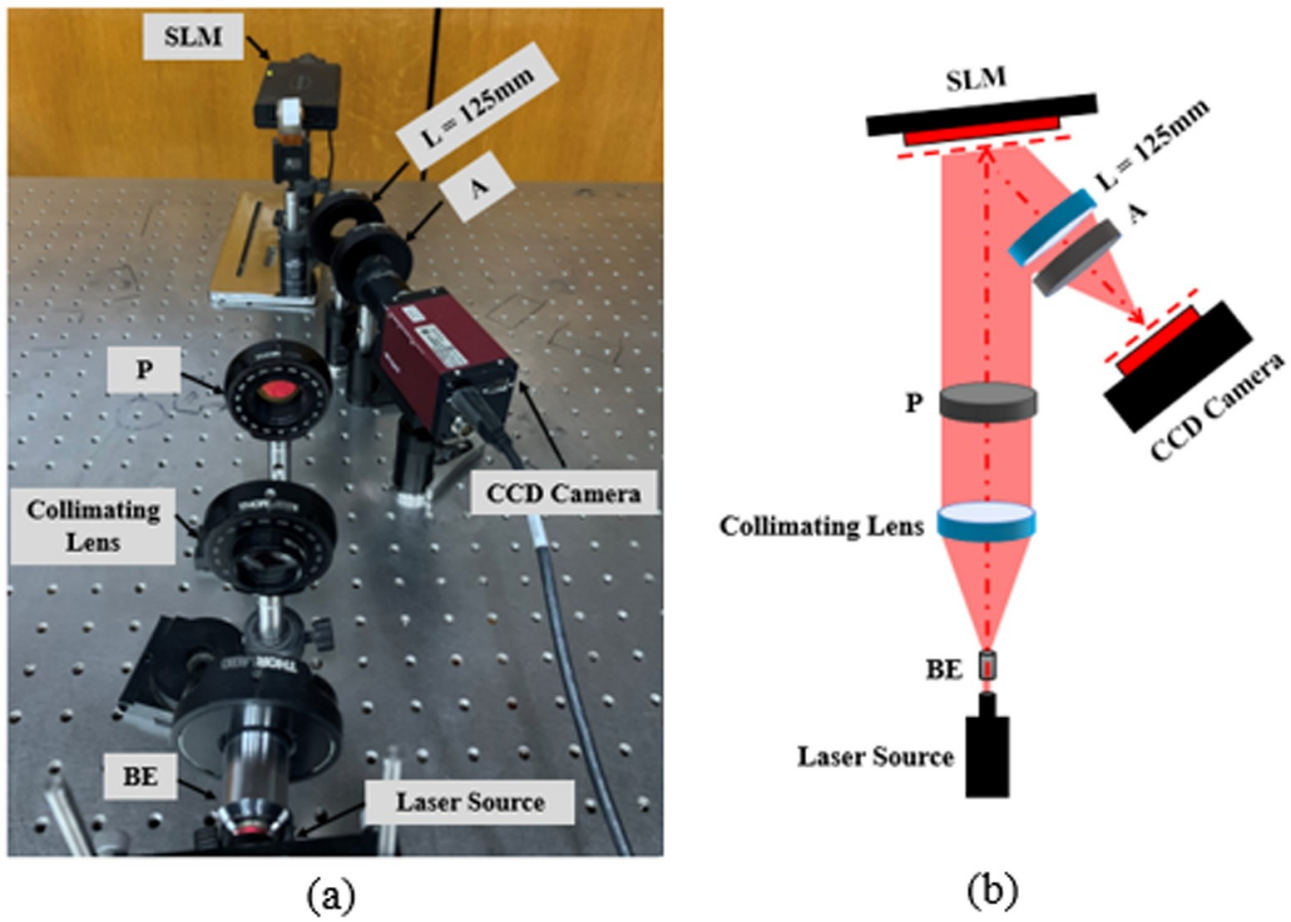
Holographic projection system: (a) an experimental setup, and (b) a schematic draw.

The demonstration of the optical reconstruction system is shown in Fig 4. The used reflective phase-only SLM is provided by HoloEye and is commercially known as Pluto model. This model consists of an active liquid crystal on silicon (*LCOS*) matrix with sampling interval and resolution of 8*μm* and 1920 × 1080 pixels, respectively. The SLM operates with a frame rate of 60*Hz*. It is characterized at λ = 670*nm* to realize a phase modulation in the range of [0, 2*π*]. The input laser source of a wavelength λ = 670*nm* is expanded by a beam expander (*BE*) and collimated to illuminate the SLM surface with a plane wave. The SLM is placed at the front focal plane of a Fourier lens (*L* = 125*mm*). As the SLM is a birefringent modulator, a polarizer (*P*) and analyzer (*A*) are placed before and after it to let only the fully modulated beam which corresponds to its slow axis passing to the camera. A charged-coupled device (*CCD*) camera sensor (model: Pike *F*_−_505*B*, pixel pitch of 3.345*μm* and resolution of 2452 × 2054 pixels) is placed at the back focal plane of the lens (*L*) to capture the reconstructed images.

## Results and discussion

To estimate the functional utility, validity and the robustness of our proposed system, we performed evaluations in two stages. The first stage was included within the design of the phase holograms which was started from the used automated segmentation algorithm and ended by evaluating the designed holograms. The segmentation accuracy of the proposed method was measured by computing the Sørensen-Dice similarity coefficient [56, 57] and BF score [58] between our segmentation results and the ground truth segmentation results provided by [40, 41]. Then, the root mean square error (*RMSE*) which calculates the difference between the input image (reference image) and the output image obtained from the numerical reconstruction of the phase distribution after the convergence of IFTA was calculated. Thus, *RMSE* was used to determine the optimal number of iterations which was based on the stopping criterion. We found that *ϵ* ≤ 0.05 radians gave satisfactory results. In the second stage, we evaluated the holographic projection results, i.e. the optical reconstruction results. For quantitatively assessment of the quality of the optically reconstructed images, the scaled signal to noise ratio (*SNR*), standard deviation (*σ*), and the speckle contrast ratio (*C*) were used.

### Segmentation results

**Fig 5:**
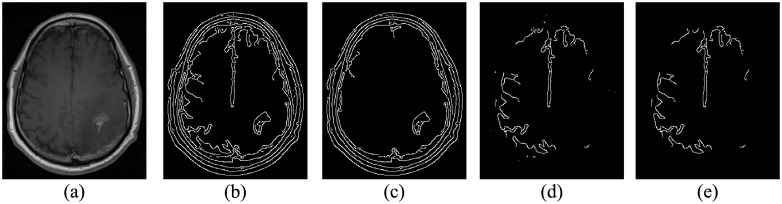
Automatic set of initialized target points within the brain tissue: (a) an axial MR slice, (b) and (c) the result of edge detection with threshold values of 0.1 and 0.2, respectively, (d) subtraction of the image in (c) from the image in (b), and (e) the obtained initialized target points after morphological by opening operation.

Here, the segmentation results of our automated segmentation algorithm are presented. First, the FMM algorithm is started by the automated generation of the initialized target points within the brain tissue based on the Canny edge detector. Regarding the Canny edge detector, the contours of all structures having some differences to the background are detected according to the selected two threshold values. Given that the skull and tumor area have higher contrast than the brain tissue, their contours would appear along with the brain tissue when concerning it. Accordingly, we apply the Canny edge detector to the MR image twice with two high thresholds values of 0.1 and 0.2 which gave good results in all MR exams of the datasets. At the same time, the lower threshold values is set to zero in both cases to preserve all the weak points in connection to the detected strong points. When the MR image was threshold by 0.1 value, the contours of the skull, tumor area and brain tissue were detected. While, when a higher threshold value of 0.2 was chosen, most of the brain tissue contours vanished. The subtraction of the two threshold results followed by morphological by opening operation [59], to remove the small connected components that might result in noise, gave the initialized target points within the brain tissue contour. The steps for the automatic setting of the initialized points within the brain tissue are presented in Fig 5. Second, the initialized target point within the tumor area was set to be the point of the highest gray-level intensity value in the MR image after skull stripping.

**Fig 6:**
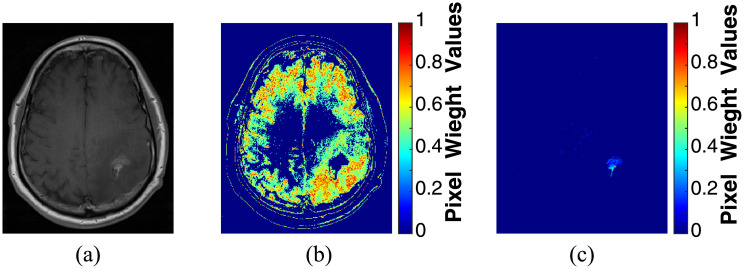
Pixel weight matrices calculation: (a) an axial MR slice, (b) pixel weight matrix of the brain tissue, and (c) pixel weight matrix of the tumor area.

**Fig 7:**
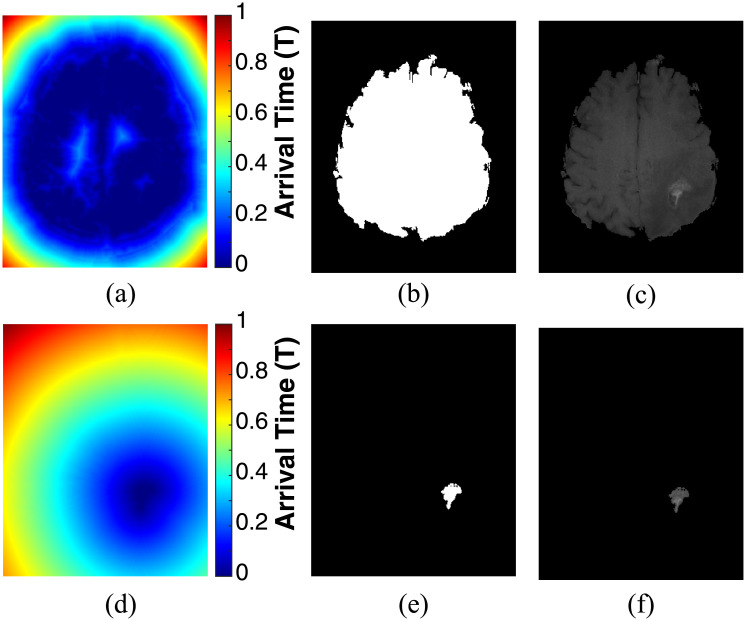
Application of FMM on the MRI slice shown in Fig 5(a): (a) arrival time estimation for the brain tissue, (b) segmented binary brain tissue, (c) segmented gray-level intensity brain tissue, (d) arrival time estimation for the tumor area, (e) segmented binary tumor area, and (f) segmented gray-level intensity tumor area.

Consequently, two matrices containing the weight values for each pixel in the brain tissue and the tumor area were generated from the MR slice shown in Fig 6(a). The result of this step is shown in Fig 6(b) and 6(c). It is concluded from the obtained results, that the pixel weight values for the brain tissue are completely different from the tumor area since the pixel weight matrices were implemented with respect to the intensity values of regions of interest. As it was mentioned above, the FMM is a quick approach utilized to solve the Eikonal equation (Eq (1)) [53] by considering the propagation of a front defined by a group of seed points set within the areas of interest. Hence, the newly implemented matrices shown in Fig 6(b) and 6(c) were considered as an initial estimate of the brain tissue and tumor area, respectively. In addition, they were conducted to the FMM approach as the speed function *F* for estimating the Function *T*. Here, the speed function is defined by the color of the pixels in the pixel weight matrices in which a bright pixel, having high weight values result in a higher speed and vice versa. Thus, the speed in the interested regions will be the highest. The arrival time is estimated and presented in Fig 7(a) and 7(d) for the brain tissue and tumor area, respectively. By thresholding the arrival time matrix (a value of 0.009 gave good results in all MR exams of the datasets), the segmented binary and gray-level intensity brain tissue and tumor area are shown in Fig 7(b), 7(c), 7(e) and 7(f), respectively.

**Fig 8:**
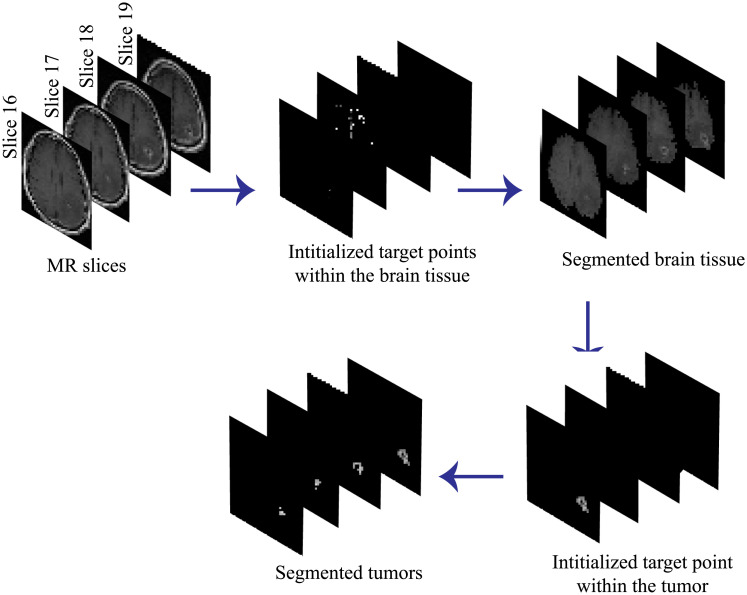
The process of the proposed automated segmentation algorithm.

The proposed automated segmentation method to extract the brain tissue and tumor volume from the four MR slice in which the tumor appears is illustrated in Fig 8.

**Fig 9:**
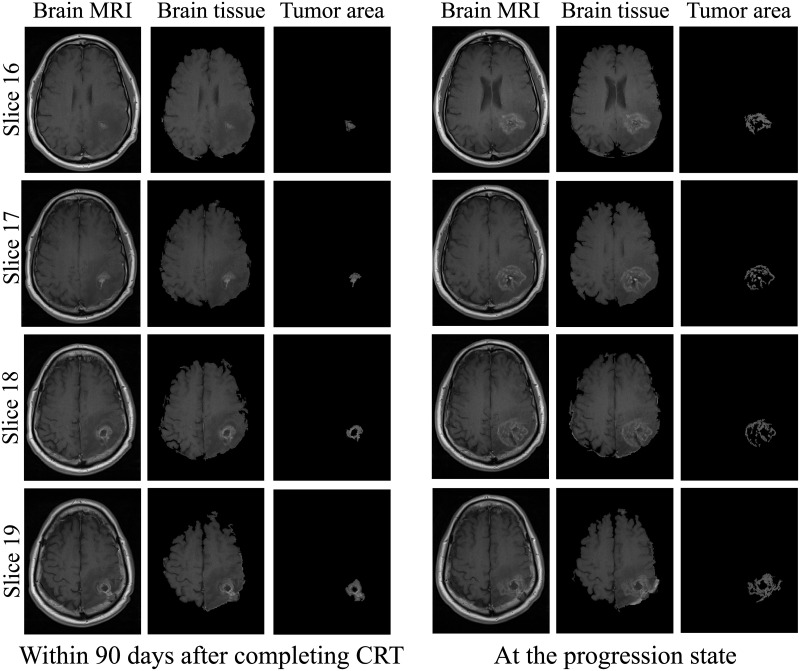
Segmentation of brain tissue and tumor areas for case 1.

**Fig 10:**
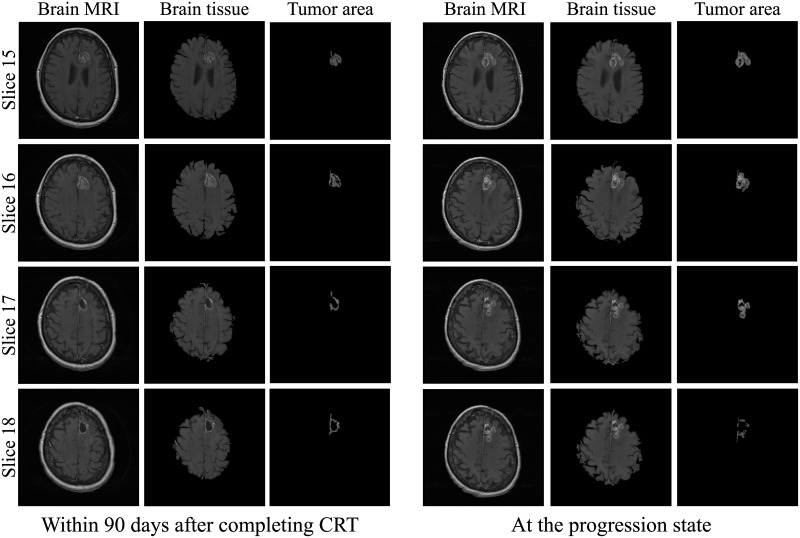
Segmentation of brain tissue and tumor areas for case 2.

**Fig 11:**
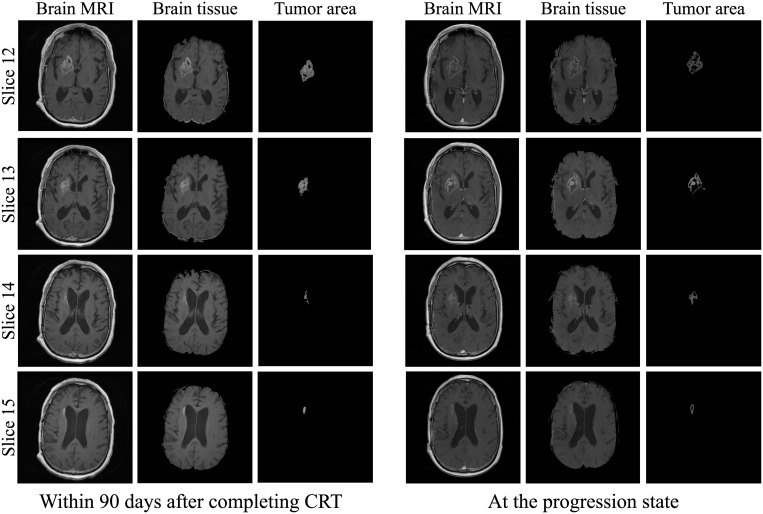
Segmentation of brain tissue and tumor areas for case 3.

For the results of this study, three cases of patients suffering from glioblastoma were documented. For each patient, two MR exams were collected: within ninety days after completing CRT and at the progression state as shown in Figs 9–11.

The Sørensen-Dice similarity coefficient [56, 57], and the BF score [58] have been widely used as scalar metrics, in the range [0, 1], to evaluate the segmentation accuracy. Whereas, Dice coefficient measures the intersection between a predicted segmented region and a ground truth segmented region. While, the BF score computes how close the predicted segmented boundary matches the ground truth segmented boundary. Therefore, these two metrics were applied to measure the segmentation accuracy of our results. To achieve that, the segmented brain tumor datasets provided by [40, 41] and evaluated by an advisory group, were used as a ground truth. The obtained average values of Dice coefficient, and the BF score were 0.93, and 0.95, respectively. Theses results reveal an extremely high overlap between our segmentation results and the ground truth. Moreover, the segmentation method was very fast with an average execution time of 0.57 and 0.61 sec, for image sizes of 320 × 260 pixels and 512 × 512 pixels, respectively, to segment the brain tissue and tumor volume from the four MR slices.

### Optical reconstruction results

#### Speckle noise reduction

**Fig 12:**
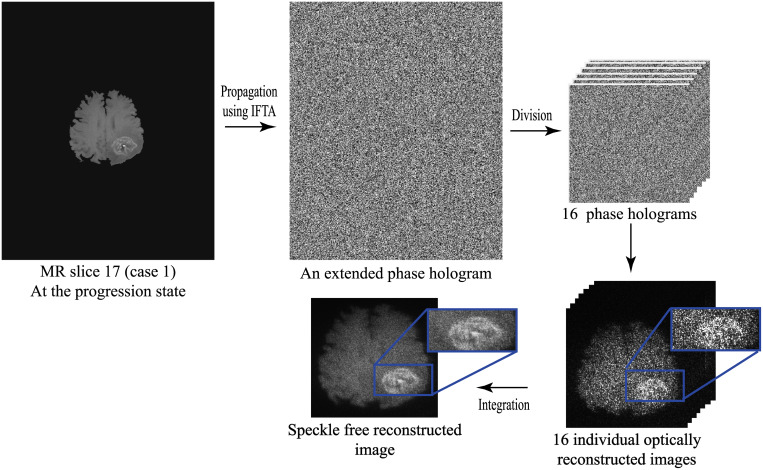
Speckle reduction procedure using temporal multiplexing of spatial frequencies.

From the sate of the art [13, 14, 36, 55], it is known that the optical reconstruction of a phase-only hologram is noisy. To overcome the speckle noise which is a common problem in displaying CGHs, a speckle reduction method proposed by Agour et. al. [13] which is based on temporal multiplexing of spatial frequencies was applied. The algorithm is illustrated in Fig 12, where the size of MR slice was 320 × 260 pixels. The procedure started by zero-padding the MR slice to be four times the size of the SLM window (1920 × 1080 pixels). The zero-padding gives a matrix with the size of 7680 × 4320 pixels and is used to be the input pattern to the AIFTA. Then, the obtained extended phase distribution was divided into a set of sixteen equally holograms each of them had the size of 1920 × 1080 pixels. The whole set of the sixteen holograms were displayed sequentially on the SLM. Whereas the SLM modulates the incident plane wave to generate a projected intensity at the observation plane, each separate phase distribution generates a projected intensity with different features of speckle. Utilizing the property of temporal-multiplexing of the SLM, the incoherent superposition of the sixteen reconstructed intensities is effectively suppress the speckle noise across the observation plane. As the switching time of the SLM was 50 msec, the whole time required for projecting the sixteen phase holograms and capturing the reconstructed images was 0.8 sec.

The signal to noise ratio (*SNR*) and the standard deviation (*σ*) were used to quantitatively evaluate the quality of the reconstructed images. The scaled *SNR* can be calculated from [13, 60]:

$$\begin{array}{c} SNR=\frac{{∥I∥}^{2}}{∥I-\beta I_{d}{∥}^{2}}, \end{array}$$ (16)

and the factor *β* is given by:

$$\begin{array}{c} \beta^{2}=\frac{{∥I∥}^{2}}{∥I_{d}{∥}^{2}}, \end{array}$$ (17)

where *I* and *I*_*d*_ are the predefined projected intensity (the original MR image) and the light distribution of the optically projected intensity that was recorded by the CCD, respectively, and ‖.‖ denotes the square norm. The factor *β* was used to ensure that the total intensity difference didn’t affect the value of the *SNR*. The value of the *SNR* for the regions inside the rectangle area in Fig 12 were *SNR*_1_ = 1.36 and *SNR*_16_ = 2.47, before and after applying the speckle reduction algorithm, respectively. As well as, the standard deviation of the same regions were *σ*_1_ = 74.91 and *σ*_16_ = 43.50, before and after applying the speckle reduction algorithm, respectively. This result shows a good agreement with the improvement published in [13].

Moreover, the speckle contrast (*C*) was used to evaluate the speckle noise suppression, it was calculated by dividing the standard deviation (*σ*) of an optically reconstructed image by its mean (*μ*) [26, 61] as:

$$\begin{array}{c} C=\frac{\sigma }{\mu }, \end{array}$$ (18)

*C* was measured for the reconstructed images from one and sixteen holograms, it was *C*_1_ = 0.733 and *C*_16_ = 0.405, respectively. The lower values of *C* indicates better reconstruction results [26, 61] and verifies the suppression of the speckle noise. All of the obtained results showed a significant quality improvement of the optically reconstructed images due to the reduction of the speckle noise effect.

#### Three-dimensional visualization map of the tumor

**Fig 13:**
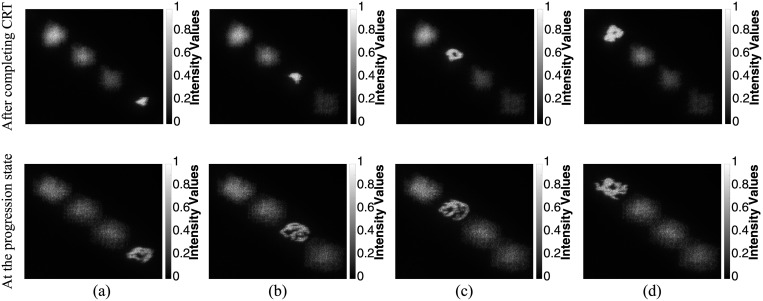
3D optical reconstruction of the tumor areas from 4 sequential slices for case 1 in Fig 9 after completing CRT and at the progression state focusing on the tumor area of: (a) slice 16, (b) slice 17, (c) slice 18, and (d) slice 19.

Fig 13 shows the 3D optical reconstruction results of the first scheme that displays the tumor’s 3D visualization maps of case 1 in Fig 9: after completing CRT and at the progression state “Video in S1 Video”, respectively. In this scheme, the 3D optical reconstruction was achieved through different layers which helps to visualize the volume of the tumor in each state. Furthermore, the properties of the tumors’ volume were calculated for each state. The volume of the tumor was 51 *cm*^3^ and 104 *cm*^3^ after completing CRT and at the progression state, respectively. The solidity was 0.53 and 0.39 after completing CRT and at the progression state, respectively.

Therefore, by integrating the 3D visualization scheme with the quantitative analysis of the tumor volume, the evaluation of the extent of response to the treatment method could be accurately achieved.

#### Comparative holographic projection results

**Fig 14:**
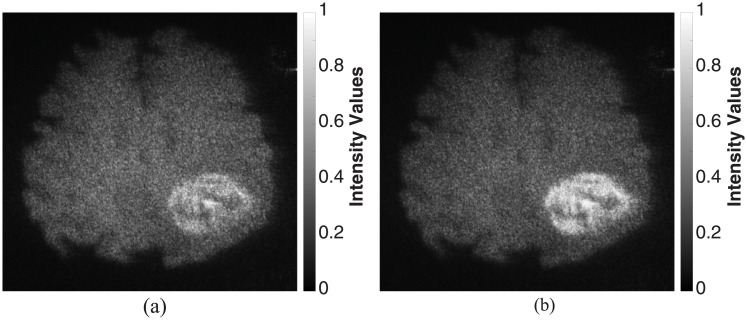
Optical reconstruction results of brain tissue: (a) and (b) before and after intensity manipulation of the detected tumor’s hologram, respectively. The physical size of the image was 3.211 × 2.609*μm*^2^.

The reconstructed brain tissue before and after the intensity manipulation of the tumor’s hologram is presented in Fig 14. The achieved results indicate that there is a significant difference between the intensity of the tumor areas in Fig 14(a) and 14(b) that could increase the ability of detecting fine tumor progression by highlighting it.

**Fig 15:**
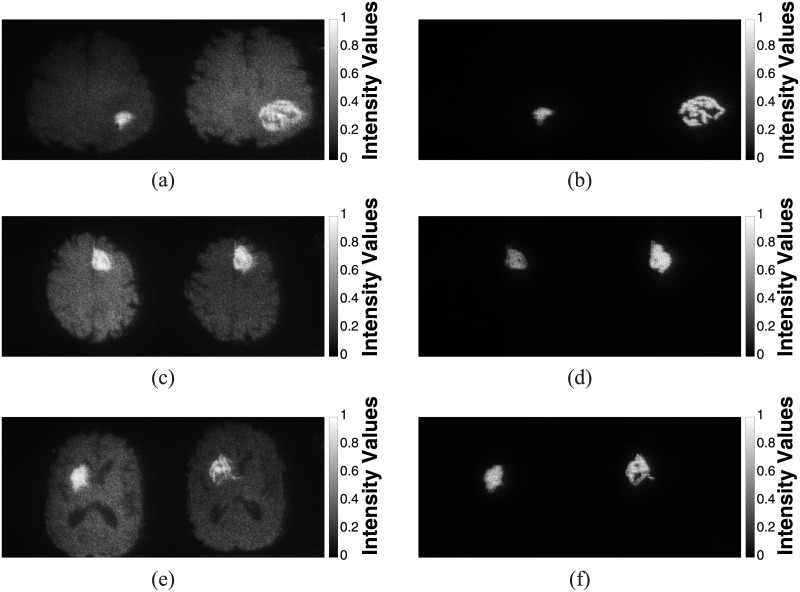
Comparative holographic projection of follow-up MR exams for slices 17, 16 and 13 of three cases in Figs 9–11, respectively: (a), (c) and (e) for the brain tissue (with highlighted tumor area) after completing CRT (left image) and at the progression state (right image). (b), (d) and (f) for the tumor areas after completing CRT (left image) and at the progression state (right image).

The comparative holographic projection results of the second scheme for selected slices of the three cases of glioblastoma patients presented in Figs 9–11 are demonstrated in Fig 15 showing the follow-up exams alongside. Fig 15(a) shows the brain tissue with highlighted tumor area for case 1 in Fig 9, after completing CRT (left image) and at the progression state (right image). Fig 15(b) shows only comparative holographic projection of the tumor area for the same slice, after completing CRT (left image) and at the progression state (right image). It is obvious from Fig 15(b) that the tumor area after completing CRT shows an irregular small lesion, while at the progression state the lesion shows an increase of the tumor’s margin indicating that there is a progression. Moreover, the tumor area and solidity were measured in each state, they were 1.64 *cm*^2^, 4.86 *cm*^2^ and 0.69, 0.39 after completing CRT and at the progression state, respectively.

For the second case in Fig 10, the comparative holographic projection results are presented in Fig 15(c) and 15(d). In Fig 15(d), the tumor area after completing CRT shows an irregular lesion, its area and solidity were 3.36 *cm*^2^ and 0.66, respectively (left image), whereas a small increase in the tumor area and solidity (area = 5.49 *cm*^2^, solidity = 0.75) were occurred at the progression state (right image).

Finally, for case 3 in Fig 11, the comparative holographic projection results are shown in Fig 15(e) and 15(f) “Video in S2 Video”. In Fig 15(f), the tumor area and solidity after completing CRT were 3.96 *cm*^2^ and 0.74, respectively, whereas at the progression state (right image) the area and solidity decreased (area = 3.57 *cm*^2^, solidity = 0.45).

The visual comparison shown in Fig 15 was utilized for demonstrating how easily the comparative process helps for the evaluation and the assessment of each state.

## Conclusions

In this work, a new comparative holographic projection system for visual and quantitative assessment of tumor progression for glioblastoma patients was proposed and its effectiveness was demonstrated by optical experiments. At the beginning, the brain tissue and the brain tumor areas were precisely segmented from the magnetic resonance (MR) slice using the fast marching method (FMM) which was implemented on a computed pixel weight matrix based on an automatic detection of the initialized target points. The evaluation of our automated segmentation algorithm was performed by comparing our segmentation results with the segmentation results obtained by [40, 41] as a ground truth. The accuracy was 0.93 using Sørensen-Dice similarity coefficient, and 0.95 using BF score. The average execution time to segment the brain tissue and tumor volume from the four MR slices was 0.57 and 0.61 sec, for the MR images of size 320 × 260 pixels and 512 × 512 pixels, respectively. Thus, the proposed algorithm was accurate and very fast. Then, segmented structures’ holograms were calculated and optimized based on an adaptive iterative Fourier transform algorithm (AIFTA) that is analogue to the well-known Gerchberg Saxton (GS) algorithm. The designed phase holograms were evaluated using the root mean square error (*RMSE*) which was used to monitor the quality of the numerical reconstructed images, and to determine the optimal number of iterations based on the suggested stopping criterion. The optimal number of iterations was 162, for an extended hologram of size 7680 × 4320 pixels, with calculation time 492 sec using Intel Core i5-3210 CPU 2.5 GHz with 4 GB RAM. However, this time could be significantly decreased in milliseconds range if the approach was paralyzed with a GPU. Moreover, the calculated phase holograms were modified to represent two different reconstruction schemes. The first scheme displayed a 3D visualization map of the tumor areas that were segmented from sequential MR slices. Whereas, the second one provided the follow-up images alongside highlighting the tumor areas for efficient visualization of its progression in the observation plane. Within the optical reconstruction, temporal multiplexing of spatial frequencies was applied to suppress the speckle noise and to get improved reconstructed images. In both schemes, all modified phase-holograms were added to provide a single 2D hologram that was uploaded on a reflective phase-only spatial light modulator (SLM) for optical reconstruction. The whole process of displaying the phase holograms and capturing the reconstructed images took less than 1 sec, since the switching time of the used SLM was 50 msec. The optical reconstruction results were evaluated using the scaled signal to noise ratio (*SNR*), standard deviation (*σ*), and the speckle contrast (*C*). The obtained results revealed a significant quality improvement of the optically reconstructed images. The experimental results exhibited that the proposed projection system can be used as an effective optical method for 3D visualization of the tumor and for displaying two follow-up MR exams in a side-by-side mode highlighting the tumor areas to evaluate the disease progression. Furthermore, this optical display method may be valuable tool in clinical trails and can lead to development of new treatment approaches.

## Supporting information

S1 Video 3D visualization of brain tumor progression for case 1: At the progression state. (AVI)

S2 Video Comparative holographic projection results for slice 13 of case 3: After completing CRT and at the progression state. (AVI)
