# Correction: Three-dimensional visualization of brain tumor progression based accurate segmentation via comparative holographic projection

**DOI:** 10.1371/journal.pone.0251614

**Published:** 2021-05-10

**Authors:** The PLOS ONE staff

Fig 8 is incorrect. The authors have provided a corrected version here. The publisher apologizes for the error.

**Fig 8:**
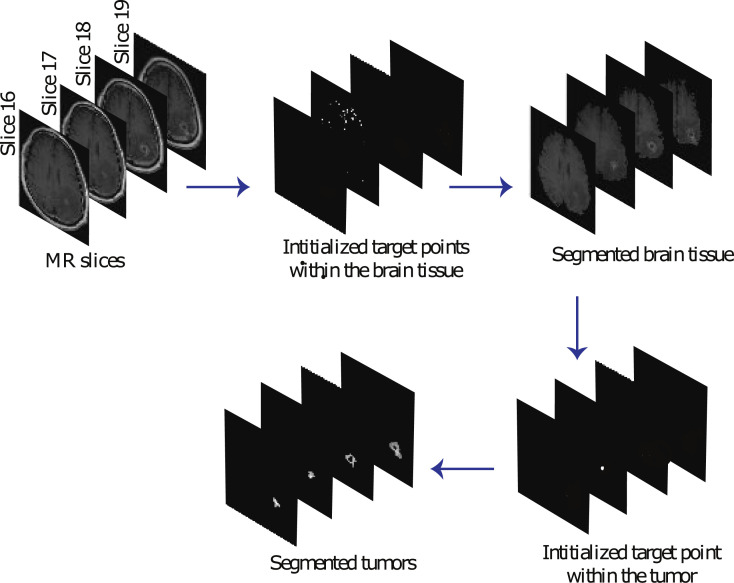
The process of the proposed automated segmentation algorithm.
